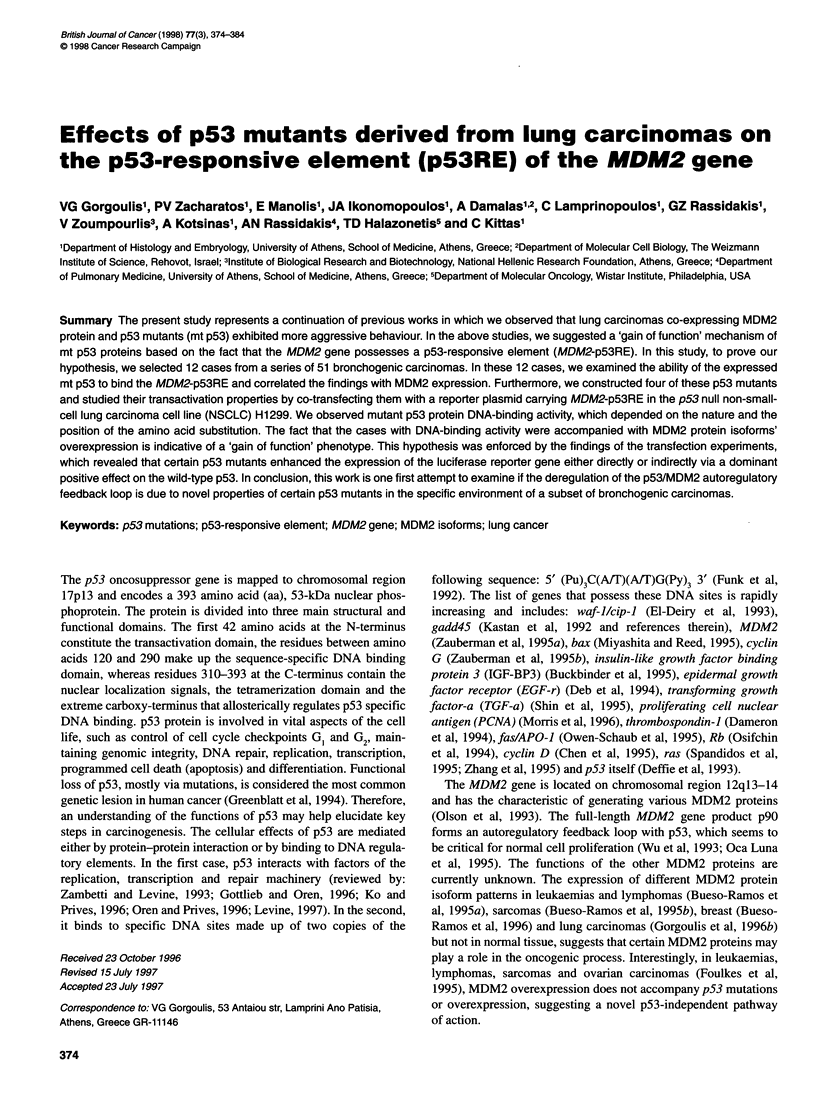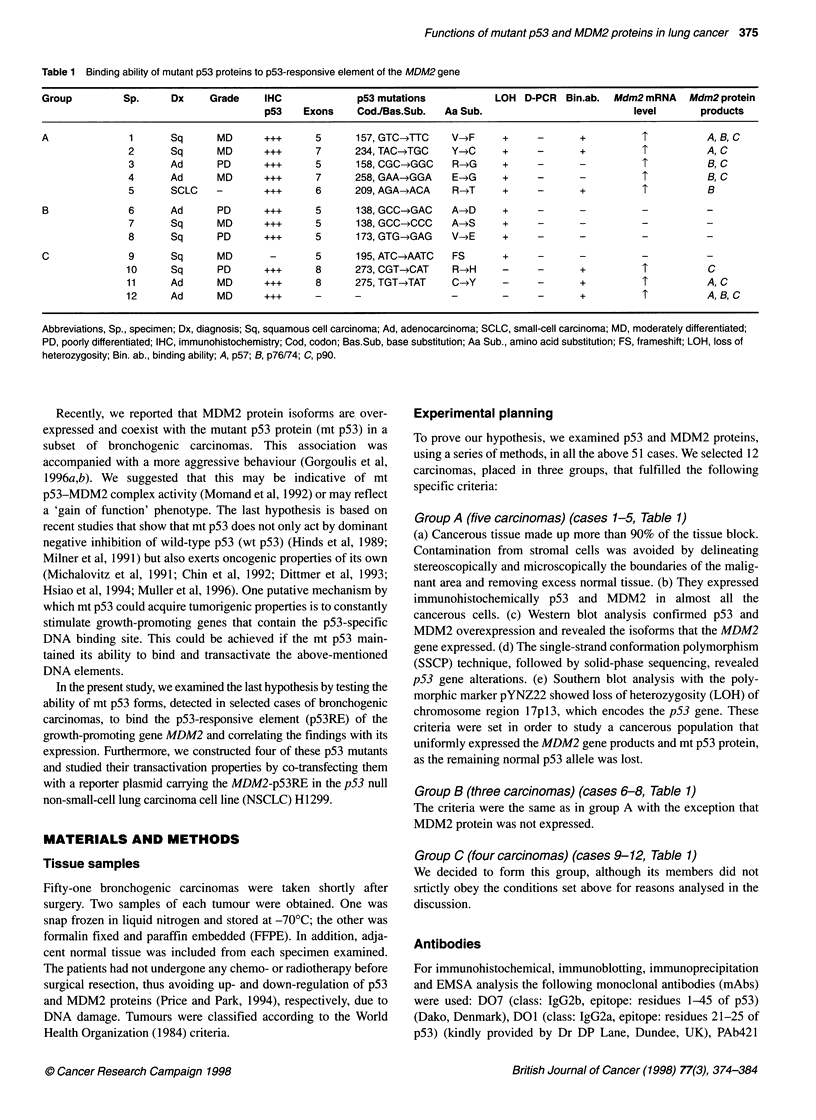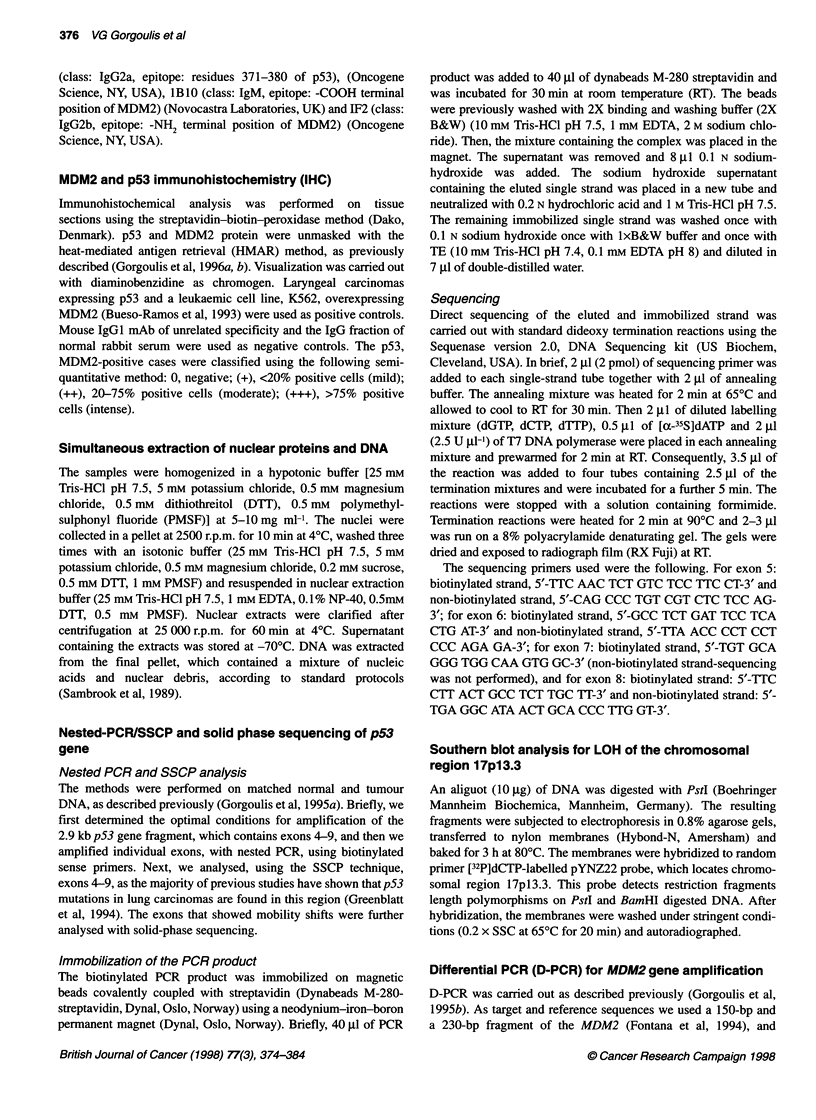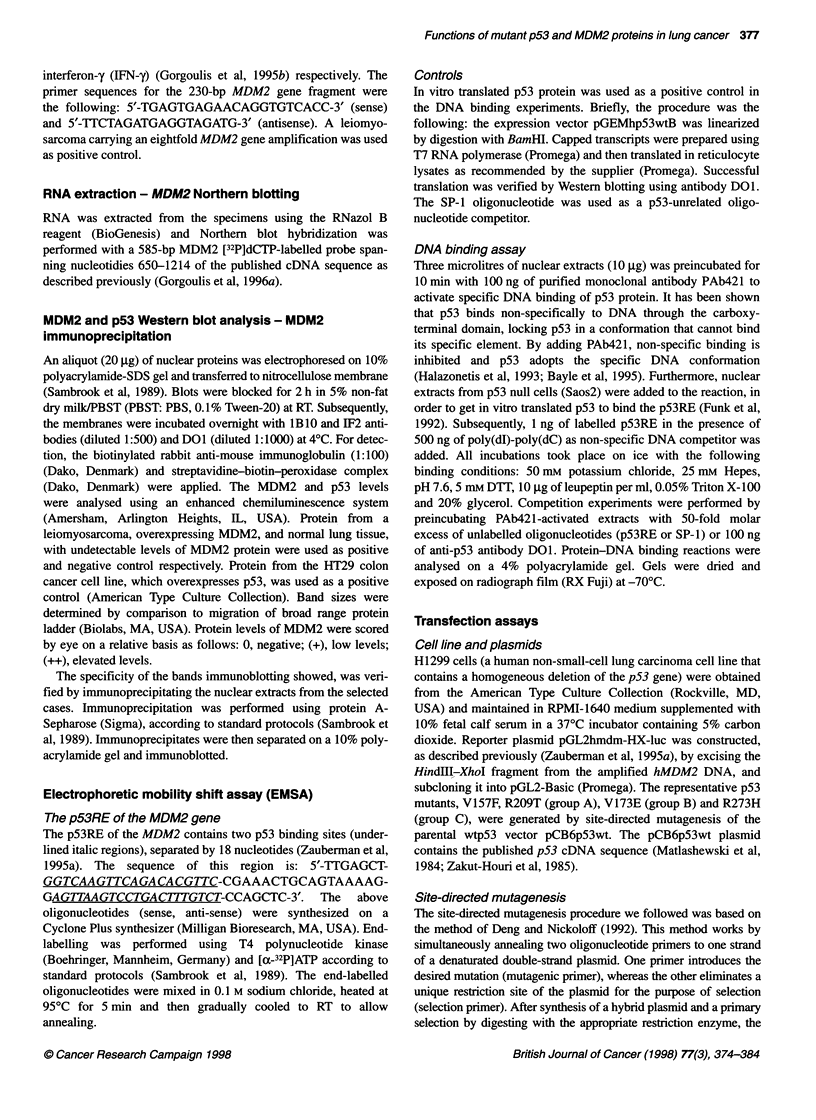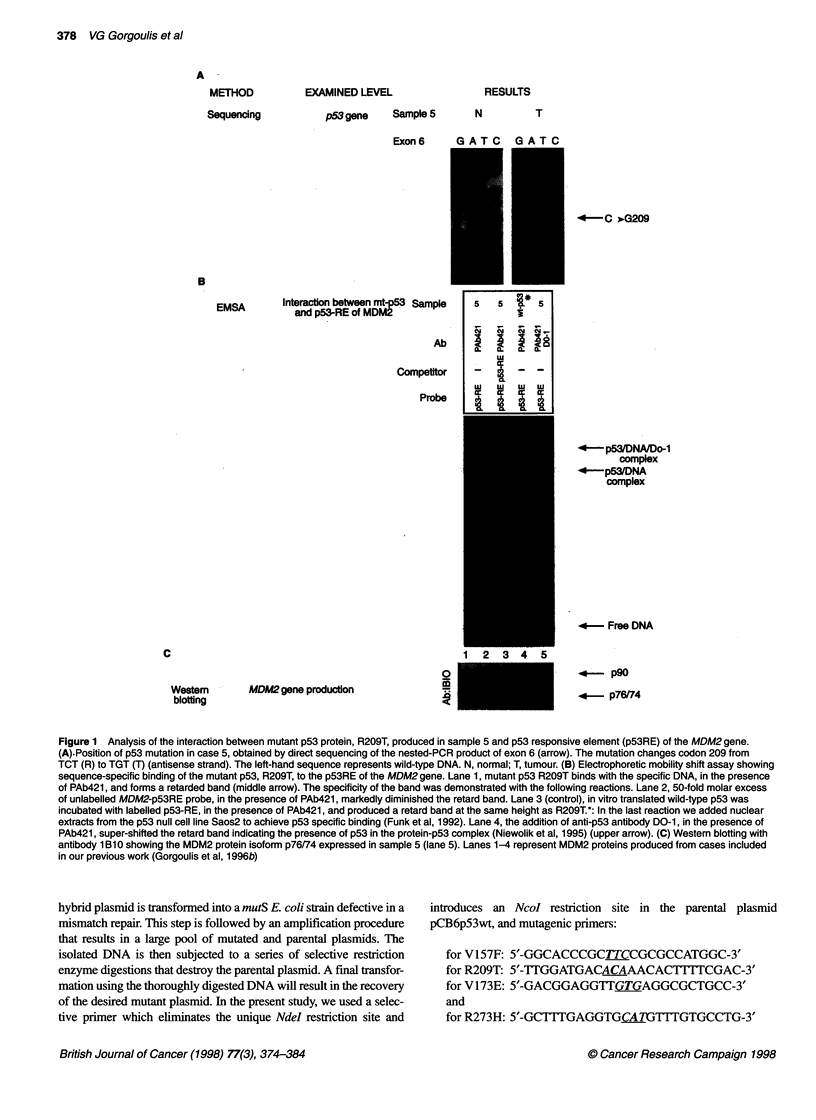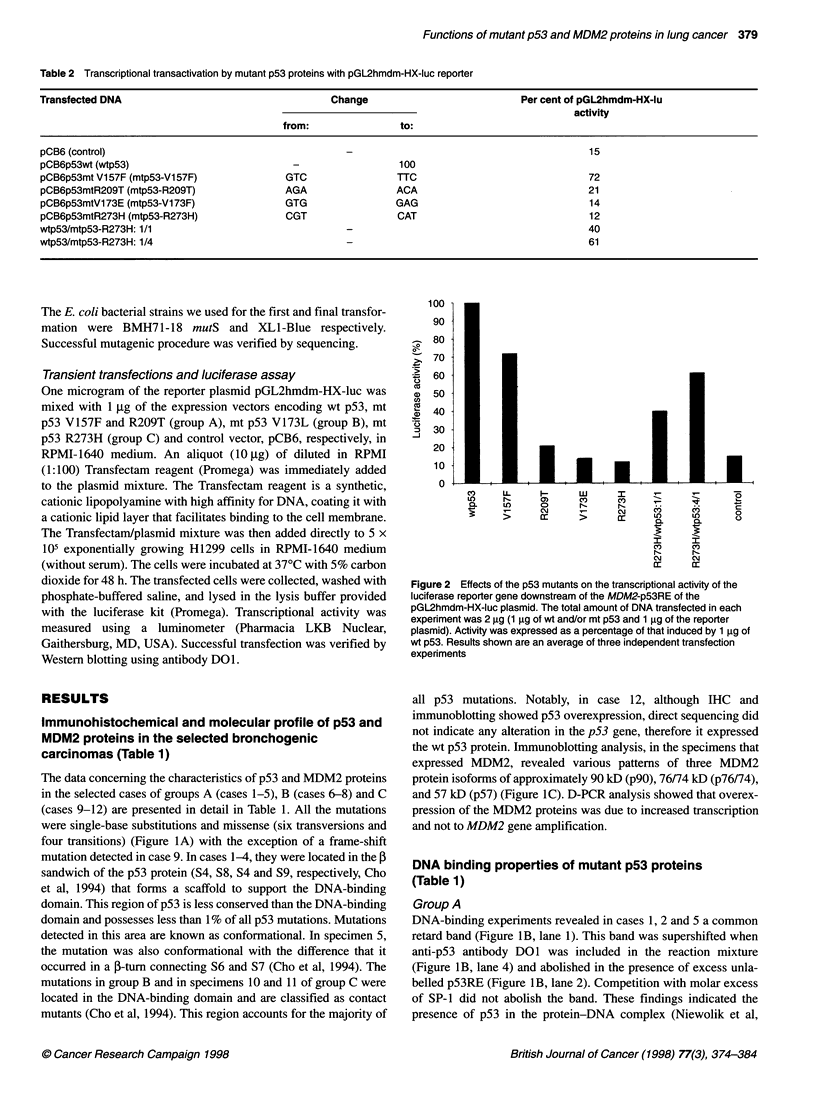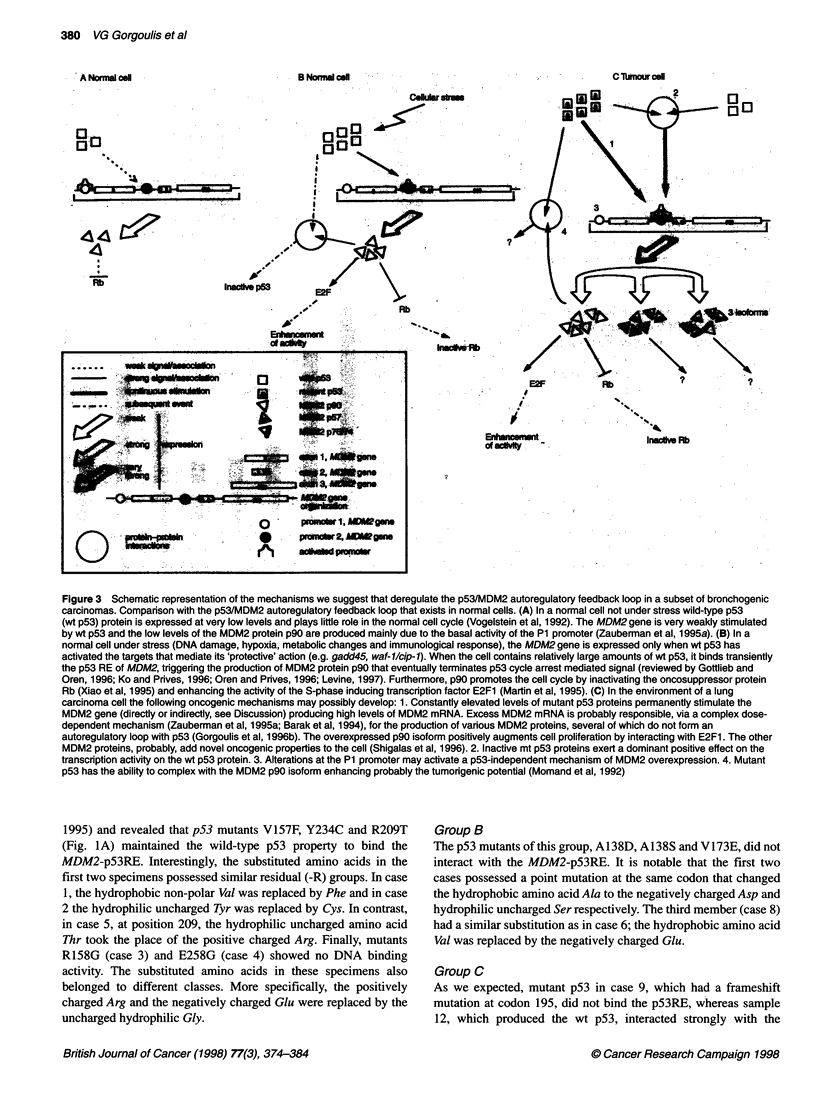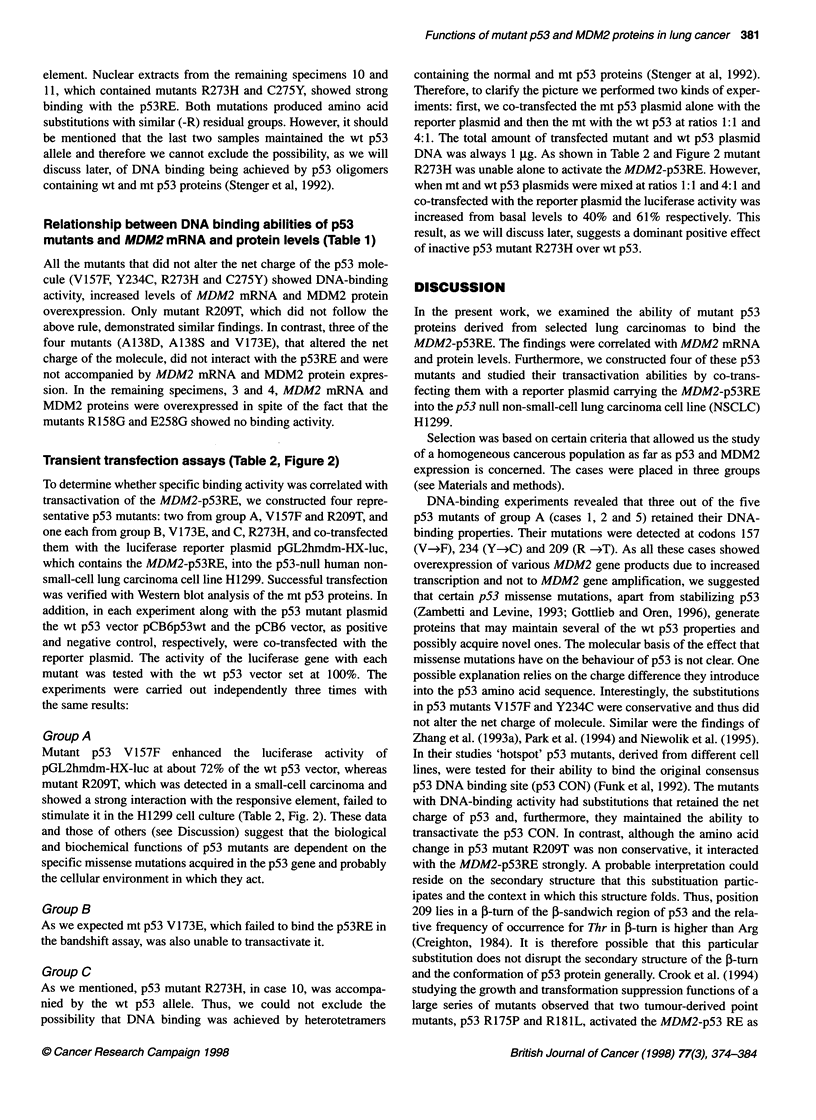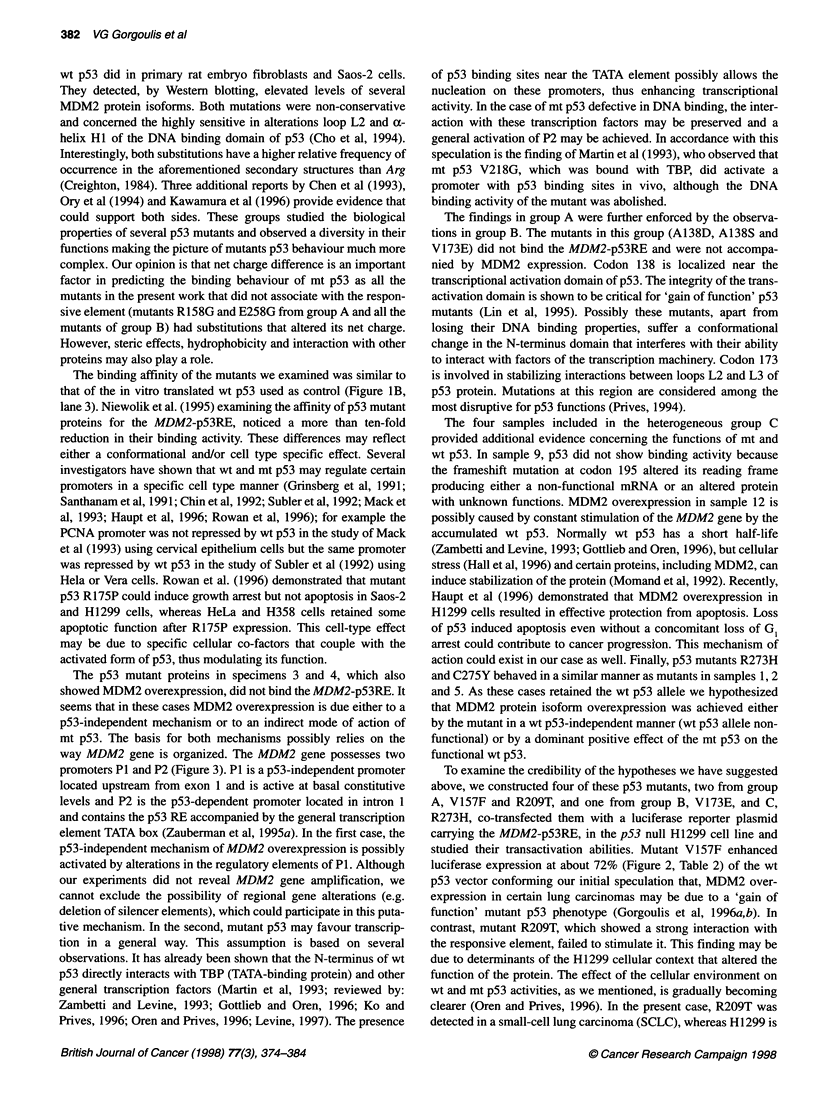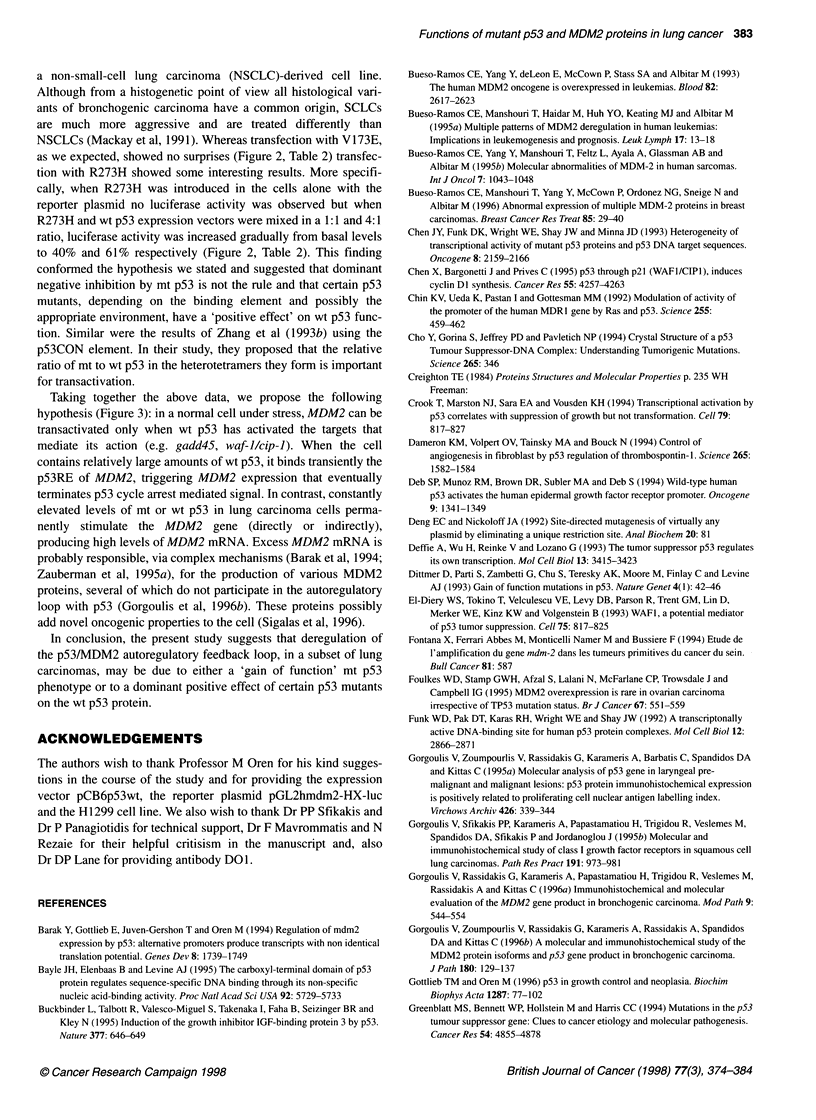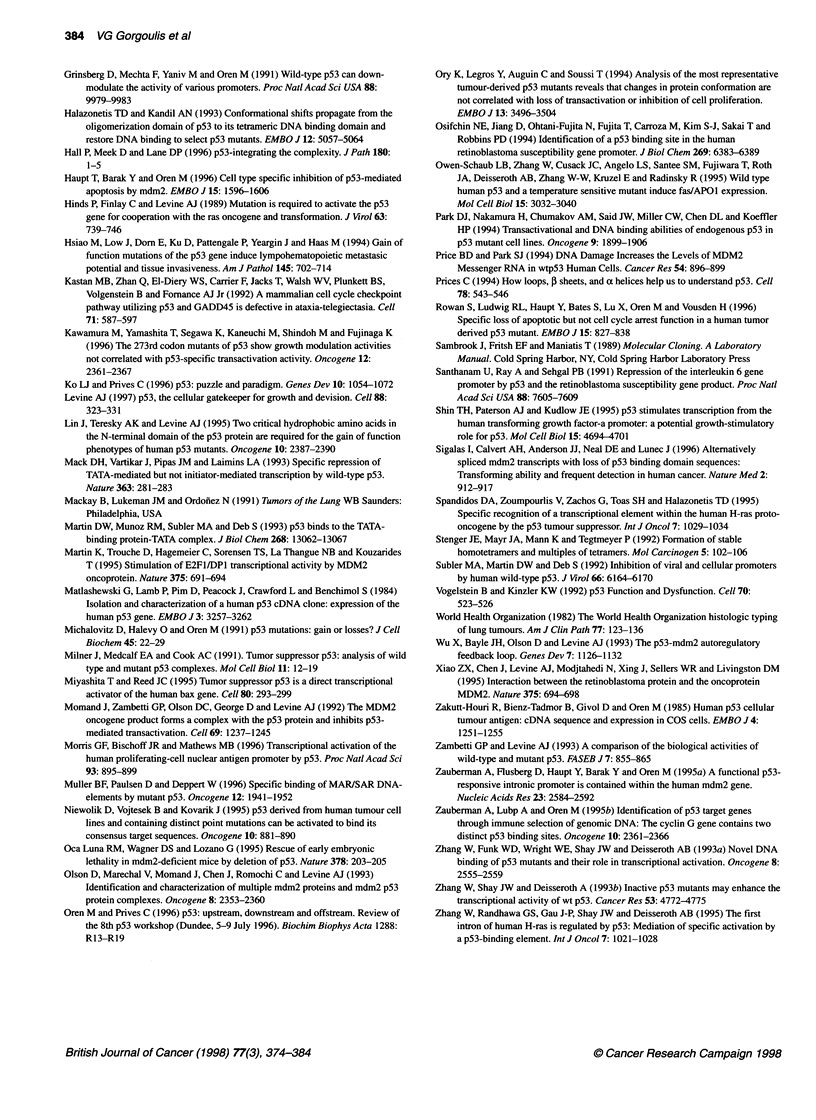# Effects of p53 mutants derived from lung carcinomas on the p53-responsive element (p53RE) of the MDM2 gene.

**DOI:** 10.1038/bjc.1998.60

**Published:** 1998

**Authors:** V. G. Gorgoulis, P. V. Zacharatos, E. Manolis, J. A. Ikonomopoulos, A. Damalas, C. Lamprinopoulos, G. Z. Rassidakis, V. Zoumpourlis, A. Kotsinas, A. N. Rassidakis, T. D. Halazonetis, C. Kittas

**Affiliations:** Department of Histology and Embryology, University of Athens, School of Medicine, Greece.

## Abstract

**Images:**


British Journal of Cancer (1998) 77(3), 374–384
© 1998 Cancer Research Campaign

### Effects of p53 mutants derived from lung carcinomas on the p53-responsive element (p53RE) of the *MDM2* gene

**VG Gorgoulis[1], PV Zacharatos[1], E Manolis[1], JA Ikonomopoulos[1], A Damalas[1,2], C Lamprinopoulos[1], GZ Rassidakis[1], V Zoumpourlis[3], A Kotsinas[1], AN Rassidakis[4], TD Halazonetis[5] and C Kittas[1]**

[1]Department of Histology and Embryology, University of Athens, School of Medicine, Athens, Greece; [2]Department of Molecular Cell Biology, The Weizmann Institute of Science, Rehovot, Israel; [3]Institute of Biological Research and Biotechnology, National Hellenic Research Foundation, Athens, Greece; [4]Department of Pulmonary Medicine, University of Athens, School of Medicine, Athens, Greece; [5]Department of Molecular Oncology, Wistar Institute, Philadelphia, USA

**Summary** The present study represents a continuation of previous works in which we observed that lung carcinomas co-expressing MDM2 protein and p53 mutants (mt p53) exhibited more aggressive behaviour. In the above studies, we suggested a 'gain of function' mechanism of mt p53 proteins based on the fact that the *MDM2* gene possesses a p53-responsive element (*MDM2*-p53RE). In this study, to prove our hypothesis, we selected 12 cases from a series of 51 bronchogenic carcinomas. In these 12 cases, we examined the ability of the expressed mt p53 to bind the *MDM2*-p53RE and correlated the findings with MDM2 expression. Furthermore, we constructed four of these p53 mutants and studied their transactivation properties by co-transfecting them with a reporter plasmid carrying *MDM2*-p53RE in the *p53* null non-small-cell lung carcinoma cell line (NSCLC) H1299. We observed mutant p53 protein DNA-binding activity, which depended on the nature and the position of the amino acid substitution. The fact that the cases with DNA-binding activity were accompanied with MDM2 protein isoforms' overexpression is indicative of a 'gain of function' phenotype. This hypothesis was enforced by the findings of the transfection experiments, which revealed that certain p53 mutants enhanced the expression of the luciferase reporter gene either directly or indirectly via a dominant positive effect on the wild-type p53. In conclusion, this work is one first attempt to examine if the deregulation of the p53/MDM2 autoregulatory feedback loop is due to novel properties of certain p53 mutants in the specific environment of a subset of bronchogenic carcinomas.

**Keywords:** *p53* mutations; p53-responsive element; *MDM2* gene; MDM2 isoforms; lung cancer

The *p53* oncosuppressor gene is mapped to chromosomal region 17p13 and encodes a 393 amino acid (aa), 53-kDa nuclear phosphoprotein. The protein is divided into three main structural and functional domains. The first 42 amino acids at the N-terminus constitute the transactivation domain, the residues between amino acids 120 and 290 make up the sequence-specific DNA binding domain, whereas residues 310–393 at the C-terminus contain the nuclear localization signals, the tetramerization domain and the extreme carboxy-terminus that allosterically regulates p53 specific DNA binding. p53 protein is involved in vital aspects of the cell life, such as control of cell cycle checkpoints $G_1$ and $G_2$, maintaining genomic integrity, DNA repair, replication, transcription, programmed cell death (apoptosis) and differentiation. Functional loss of p53, mostly via mutations, is considered the most common genetic lesion in human cancer (Greenblatt et al, 1994). Therefore, an understanding of the functions of p53 may help elucidate key steps in carcinogenesis. The cellular effects of p53 are mediated either by protein–protein interaction or by binding to DNA regulatory elements. In the first case, p53 interacts with factors of the replication, transcription and repair machinery (reviewed by: Zambetti and Levine, 1993; Gottlieb and Oren, 1996; Ko and Prives, 1996; Oren and Prives, 1996; Levine, 1997). In the second, it binds to specific DNA sites made up of two copies of the following sequence: 5′ $(Pu)_3C(A/T)(A/T)G(Py)_3$ 3′ (Funk et al, 1992). The list of genes that possess these DNA sites is rapidly increasing and includes: *waf-1/cip-1* (El-Deiry et al, 1993), *gadd45* (Kastan et al, 1992 and references therein), *MDM2* (Zauberman et al, 1995*a*), *bax* (Miyashita and Reed, 1995), *cyclin G* (Zauberman et al, 1995*b*), *insulin-like growth factor binding protein 3* (IGF-BP3) (Buckbinder et al, 1995), *epidermal growth factor receptor* (*EGF-r*) (Deb et al, 1994), *transforming growth factor-a* (*TGF-a*) (Shin et al, 1995), *proliferating cell nuclear antigen (PCNA)* (Morris et al, 1996), *thrombospondin-1* (Dameron et al, 1994), *fas/APO-1* (Owen-Schaub et al, 1995), *Rb* (Osifchin et al, 1994), *cyclin D* (Chen et al, 1995), *ras* (Spandidos et al, 1995; Zhang et al, 1995) and *p53* itself (Deffie et al, 1993).

The *MDM2* gene is located on chromosomal region 12q13–14 and has the characteristic of generating various MDM2 proteins (Olson et al, 1993). The full-length *MDM2* gene product p90 forms an autoregulatory feedback loop with p53, which seems to be critical for normal cell proliferation (Wu et al, 1993; Oca Luna et al, 1995). The functions of the other MDM2 proteins are currently unknown. The expression of different MDM2 protein isoform patterns in leukaemias and lymphomas (Bueso-Ramos et al, 1995*a*), sarcomas (Bueso-Ramos et al, 1995*b*), breast (Bueso-Ramos et al, 1996) and lung carcinomas (Gorgoulis et al, 1996*b*) but not in normal tissue, suggests that certain MDM2 proteins may play a role in the oncogenic process. Interestingly, in leukaemias, lymphomas, sarcomas and ovarian carcinomas (Foulkes et al, 1995), MDM2 overexpression does not accompany *p53* mutations or overexpression, suggesting a novel p53-independent pathway of action.

*Received 23 October 1996*
*Revised 15 July 1997*
*Accepted 23 July 1997*

*Correspondence to:* VG Gorgoulis, 53 Antaiou str, Lamprini Ano Patisia, Athens, Greece GR-11146

**Table 1** Binding ability of mutant p53 proteins to p53-responsive element of the *MDM2* gene

| Group | Sp. | Dx | Grade | IHC p53 | Exons | p53 mutations Cod./Bas.Sub. | Aa Sub. | LOH | D-PCR | Bin.ab. | *Mdm2* mRNA level | *Mdm2* protein products |
|---|---|---|---|---|---|---|---|---|---|---|---|---|
| A | 1 | Sq | MD | +++ | 5 | 157, GTC→TTC | V→F | + | – | + | ↑ | *A, B, C* |
| | 2 | Sq | MD | +++ | 7 | 234, TAC→TGC | Y→C | + | – | + | ↑ | *A, C* |
| | 3 | Ad | PD | +++ | 5 | 158, CGC→GGC | R→G | + | – | – | ↑ | *B, C* |
| | 4 | Ad | MD | +++ | 7 | 258, GAA→GGA | E→G | + | – | – | ↑ | *B, C* |
| | 5 | SCLC | – | +++ | 6 | 209, AGA→ACA | R→T | + | – | + | ↑ | *B* |
| B | 6 | Ad | PD | +++ | 5 | 138, GCC→GAC | A→D | + | – | – | – | – |
| | 7 | Sq | MD | +++ | 5 | 138, GCC→CCC | A→S | + | – | – | – | – |
| | 8 | Sq | PD | +++ | 5 | 173, GTG→GAG | V→E | + | – | – | – | – |
| C | 9 | Sq | MD | – | 5 | 195, ATC→AATC | FS | + | – | – | – | – |
| | 10 | Sq | PD | +++ | 8 | 273, CGT→CAT | R→H | – | – | + | ↑ | *C* |
| | 11 | Ad | MD | +++ | 8 | 275, TGT→TAT | C→Y | – | – | + | ↑ | *A, C* |
| | 12 | Ad | MD | +++ | – | – | – | – | – | + | ↑ | *A, B, C* |

Abbreviations, Sp., specimen; Dx, diagnosis; Sq, squamous cell carcinoma; Ad, adenocarcinoma; SCLC, small-cell carcinoma; MD, moderately differentiated; PD, poorly differentiated; IHC, immunohistochemistry; Cod, codon; Bas.Sub, base substitution; Aa Sub., amino acid substitution; FS, frameshift; LOH, loss of heterozygosity; Bin. ab., binding ability; *A*, p57; *B*, p76/74; *C*, p90.

Recently, we reported that MDM2 protein isoforms are over-expressed and coexist with the mutant p53 protein (mt p53) in a subset of bronchogenic carcinomas. This association was accompanied with a more aggressive behaviour (Gorgoulis et al, 1996*a*,*b*). We suggested that this may be indicative of mt p53–MDM2 complex activity (Momand et al, 1992) or may reflect a 'gain of function' phenotype. The last hypothesis is based on recent studies that show that mt p53 does not only act by dominant negative inhibition of wild-type p53 (wt p53) (Hinds et al, 1989; Milner et al, 1991) but also exerts oncogenic properties of its own (Michalovitz et al, 1991; Chin et al, 1992; Dittmer et al, 1993; Hsiao et al, 1994; Muller et al, 1996). One putative mechanism by which mt p53 could acquire tumorigenic properties is to constantly stimulate growth-promoting genes that contain the p53-specific DNA binding site. This could be achieved if the mt p53 maintained its ability to bind and transactivate the above-mentioned DNA elements.

In the present study, we examined the last hypothesis by testing the ability of mt p53 forms, detected in selected cases of bronchogenic carcinomas, to bind the p53-responsive element (p53RE) of the growth-promoting gene *MDM2* and correlating the findings with its expression. Furthermore, we constructed four of these p53 mutants and studied their transactivation properties by co-transfecting them with a reporter plasmid carrying the *MDM2*-p53RE in the *p53* null non-small-cell lung carcinoma cell line (NSCLC) H1299.

### MATERIALS AND METHODS

### Tissue samples

Fifty-one bronchogenic carcinomas were taken shortly after surgery. Two samples of each tumour were obtained. One was snap frozen in liquid nitrogen and stored at –70°C; the other was formalin fixed and paraffin embedded (FFPE). In addition, adjacent normal tissue was included from each specimen examined. The patients had not undergone any chemo- or radiotherapy before surgical resection, thus avoiding up- and down-regulation of p53 and MDM2 proteins (Price and Park, 1994), respectively, due to DNA damage. Tumours were classified according to the World Health Organization (1984) criteria.

### Experimental planning

To prove our hypothesis, we examined p53 and MDM2 proteins, using a series of methods, in all the above 51 cases. We selected 12 carcinomas, placed in three groups, that fulfilled the following specific criteria:

#### *Group A (five carcinomas) (cases 1–5, Table 1)*

(a) Cancerous tissue made up more than 90% of the tissue block. Contamination from stromal cells was avoided by delineating stereoscopically and microscopically the boundaries of the malignant area and removing excess normal tissue. (b) They expressed immunohistochemically p53 and MDM2 in almost all the cancerous cells. (c) Western blot analysis confirmed p53 and MDM2 overexpression and revealed the isoforms that the *MDM2* gene expressed. (d) The single-strand conformation polymorphism (SSCP) technique, followed by solid-phase sequencing, revealed *p53* gene alterations. (e) Southern blot analysis with the polymorphic marker pYNZ22 showed loss of heterozygosity (LOH) of chromosome region 17p13, which encodes the *p53* gene. These criteria were set in order to study a cancerous population that uniformly expressed the *MDM2* gene products and mt p53 protein, as the remaining normal p53 allele was lost.

#### *Group B (three carcinomas) (cases 6–8, Table 1)*

The criteria were the same as in group A with the exception that MDM2 protein was not expressed.

#### *Group C (four carcinomas) (cases 9–12, Table 1)*

We decided to form this group, although its members did not srtictly obey the conditions set above for reasons analysed in the discussion.

### Antibodies

For immunohistochemical, immunoblotting, immunoprecipitation and EMSA analysis the following monoclonal antibodies (mAbs) were used: DO7 (class: IgG2b, epitope: residues 1–45 of p53) (Dako, Denmark), DO1 (class: IgG2a, epitope: residues 21–25 of p53) (kindly provided by Dr DP Lane, Dundee, UK), PAb421

(class: IgG2a, epitope: residues 371–380 of p53), (Oncogene Science, NY, USA), 1B10 (class: IgM, epitope: -COOH terminal position of MDM2) (Novocastra Laboratories, UK) and IF2 (class: IgG2b, epitope: $-NH_2$ terminal position of MDM2) (Oncogene Science, NY, USA).

### MDM2 and p53 immunohistochemistry (IHC)

Immunohistochemical analysis was performed on tissue sections using the streptavidin–biotin–peroxidase method (Dako, Denmark). p53 and MDM2 protein were unmasked with the heat-mediated antigen retrieval (HMAR) method, as previously described (Gorgoulis et al, 1996*a, b*). Visualization was carried out with diaminobenzidine as chromogen. Laryngeal carcinomas expressing p53 and a leukaemic cell line, K562, overexpressing MDM2 (Bueso-Ramos et al, 1993) were used as positive controls. Mouse IgG1 mAb of unrelated specificity and the IgG fraction of normal rabbit serum were used as negative controls. The p53, MDM2-positive cases were classified using the following semi-quantitative method: 0, negative; (+), <20% positive cells (mild); (++), 20–75% positive cells (moderate); (+++), >75% positive cells (intense).

### Simultaneous extraction of nuclear proteins and DNA

The samples were homogenized in a hypotonic buffer [25 mM Tris-HCl pH 7.5, 5 mM potassium chloride, 0.5 mM magnesium chloride, 0.5 mM dithiothreitol (DTT), 0.5 mM polymethyl-sulphonyl fluoride (PMSF)] at 5–10 mg $ml^{-1}$. The nuclei were collected in a pellet at 2500 r.p.m. for 10 min at 4°C, washed three times with an isotonic buffer (25 mM Tris-HCl pH 7.5, 5 mM potassium chloride, 0.5 mM magnesium chloride, 0.2 mM sucrose, 0.5 mM DTT, 1 mM PMSF) and resuspended in nuclear extraction buffer (25 mM Tris-HCl pH 7.5, 1 mM EDTA, 0.1% NP-40, 0.5mM DTT, 0.5 mM PMSF). Nuclear extracts were clarified after centrifugation at 25 000 r.p.m. for 60 min at 4°C. Supernatant containing the extracts was stored at –70°C. DNA was extracted from the final pellet, which contained a mixture of nucleic acids and nuclear debris, according to standard protocols (Sambrook et al, 1989).

### Nested-PCR/SSCP and solid phase sequencing of *p53* gene

### *Nested PCR and SSCP analysis*

The methods were performed on matched normal and tumour DNA, as described previously (Gorgoulis et al, 1995*a*). Briefly, we first determined the optimal conditions for amplification of the 2.9 kb *p53* gene fragment, which contains exons 4–9, and then we amplified individual exons, with nested PCR, using biotinylated sense primers. Next, we analysed, using the SSCP technique, exons 4–9, as the majority of previous studies have shown that *p53* mutations in lung carcinomas are found in this region (Greenblatt et al, 1994). The exons that showed mobility shifts were further analysed with solid-phase sequencing.

### *Immobilization of the PCR product*

The biotinylated PCR product was immobilized on magnetic beads covalently coupled with streptavidin (Dynabeads M-280-streptavidin, Dynal, Oslo, Norway) using a neodynium–iron–boron permanent magnet (Dynal, Oslo, Norway). Briefly, 40 μl of PCR product was added to 40 μl of dynabeads M-280 streptavidin and was incubated for 30 min at room temperature (RT). The beads were previously washed with 2X binding and washing buffer (2X B&W) (10 mM Tris-HCl pH 7.5, 1 mM EDTA, 2 M sodium chloride). Then, the mixture containing the complex was placed in the magnet. The supernatant was removed and 8 μl 0.1 N sodium-hydroxide was added. The sodium hydroxide supernatant containing the eluted single strand was placed in a new tube and neutralized with 0.2 N hydrochloric acid and 1 M Tris-HCl pH 7.5. The remaining immobilized single strand was washed once with 0.1 N sodium hydroxide once with 1×B&W buffer and once with TE (10 mM Tris-HCl pH 7.4, 0.1 mM EDTA pH 8) and diluted in 7 μl of double-distilled water.

### *Sequencing*

Direct sequencing of the eluted and immobilized strand was carried out with standard dideoxy termination reactions using the Sequenase version 2.0, DNA Sequencing kit (US Biochem, Cleveland, USA). In brief, 2 μl (2 pmol) of sequencing primer was added to each single-strand tube together with 2 μl of annealing buffer. The annealing mixture was heated for 2 min at 65°C and allowed to cool to RT for 30 min. Then 2 μl of diluted labelling mixture (dGTP, dCTP, dTTP), 0.5 μl of [α-$^{35}$S]dATP and 2 μl (2.5 U $μl^{-1}$) of T7 DNA polymerase were placed in each annealing mixture and prewarmed for 2 min at RT. Consequently, 3.5 μl of the reaction was added to four tubes containing 2.5 μl of the termination mixtures and were incubated for a further 5 min. The reactions were stopped with a solution containing formimide. Termination reactions were heated for 2 min at 90°C and 2–3 μl was run on a 8% polyacrylamide denaturating gel. The gels were dried and exposed to radiograph film (RX Fuji) at RT.

The sequencing primers used were the following. For exon 5: biotinylated strand, 5′-TTC AAC TCT GTC TCC TTC CT-3′ and non-biotinylated strand, 5′-CAG CCC TGT CGT CTC TCC AG-3′; for exon 6: biotinylated strand, 5′-GCC TCT GAT TCC TCA CTG AT-3′ and non-biotinylated strand, 5′-TTA ACC CCT CCT CCC AGA GA-3′; for exon 7: biotinylated strand, 5′-TGT GCA GGG TGG CAA GTG GC-3′ (non-biotinylated strand-sequencing was not performed), and for exon 8: biotinylated strand: 5′-TTC CTT ACT GCC TCT TGC TT-3′ and non-biotinylated strand: 5′-TGA GGC ATA ACT GCA CCC TTG GT-3′.

### Southern blot analysis for LOH of the chromosomal region 17p13.3

An aliguot (10 μg) of DNA was digested with *Pst*I (Boehringer Mannheim Biochemica, Mannheim, Germany). The resulting fragments were subjected to electrophoresis in 0.8% agarose gels, transferred to nylon membranes (Hybond-N, Amersham) and baked for 3 h at 80°C. The membranes were hybridized to random primer [$^{32}$P]dCTP-labelled pYNZ22 probe, which locates chromosomal region 17p13.3. This probe detects restriction fragments length polymorphisms on *Pst*I and *Bam*HI digested DNA. After hybridization, the membranes were washed under stringent conditions (0.2 × SSC at 65°C for 20 min) and autoradiographed.

### Differential PCR (D-PCR) for *MDM2* gene amplification

D-PCR was carried out as described previously (Gorgoulis et al, 1995*b*). As target and reference sequences we used a 150-bp and a 230-bp fragment of the *MDM2* (Fontana et al, 1994), and

© Cancer Research Campaign 1998

interferon-γ (IFN-γ) (Gorgoulis et al, 1995*b*) respectively. The primer sequences for the 230-bp *MDM2* gene fragment were the following: 5′-TGAGTGAGAACAGGTGTCACC-3′ (sense) and 5′-TTCTAGATGAGGTAGATG-3′ (antisense). A leiomyosarcoma carrying an eightfold *MDM2* gene amplification was used as positive control.

### RNA extraction – *MDM2* Northern blotting

RNA was extracted from the specimens using the RNazol B reagent (BioGenesis) and Northern blot hybridization was performed with a 585-bp MDM2 [$^{32}$P]dCTP-labelled probe spanning nucleotidies 650–1214 of the published cDNA sequence as described previously (Gorgoulis et al, 1996*a*).

### MDM2 and p53 Western blot analysis – MDM2 immunoprecipitation

An aliquot (20 μg) of nuclear proteins was electrophoresed on 10% polyacrylamide-SDS gel and transferred to nitrocellulose membrane (Sambrook et al, 1989). Blots were blocked for 2 h in 5% non-fat dry milk/PBST (PBST: PBS, 0.1% Tween-20) at RT. Subsequently, the membranes were incubated overnight with 1B10 and IF2 antibodies (diluted 1:500) and DO1 (diluted 1:1000) at 4°C. For detection, the biotinylated rabbit anti-mouse immunoglobulin (1:100) (Dako, Denmark) and streptavidine–biotin–peroxidase complex (Dako, Denmark) were applied. The MDM2 and p53 levels were analysed using an enhanced chemiluminescence system (Amersham, Arlington Heights, IL, USA). Protein from a leiomyosarcoma, overexpressing MDM2, and normal lung tissue, with undetectable levels of MDM2 protein were used as positive and negative control respectively. Protein from the HT29 colon cancer cell line, which overexpresses p53, was used as a positive control (American Type Culture Collection). Band sizes were determined by comparison to migration of broad range protein ladder (Biolabs, MA, USA). Protein levels of MDM2 were scored by eye on a relative basis as follows: 0, negative; (+), low levels; (++), elevated levels.

The specificity of the bands immunoblotting showed, was verified by immunoprecipitating the nuclear extracts from the selected cases. Immunoprecipitation was performed using protein A-Sepharose (Sigma), according to standard protocols (Sambrook et al, 1989). Immunoprecipitates were then separated on a 10% polyacrylamide gel and immunoblotted.

### Electrophoretic mobility shift assay (EMSA)

### *The p53RE of the MDM2 gene*

The p53RE of the *MDM2* contains two p53 binding sites (underlined italic regions), separated by 18 nucleotides (Zauberman et al, 1995a). The sequence of this region is: 5′-TTGAGCT-*GGTCAAGTTCAGACACGTTC*-CGAAACTGCAGTAAAAG-G*AGTTAAGTCCTGACTTTGTCT*-CCAGCTC-3′. The above oligonucleotides (sense, anti-sense) were synthesized on a Cyclone Plus synthesizer (Milligan Bioresearch, MA, USA). End-labelling was performed using T4 polynucleotide kinase (Boehringer, Mannheim, Germany) and [α-$^{32}$P]ATP according to standard protocols (Sambrook et al, 1989). The end-labelled oligonucleotides were mixed in 0.1 M sodium chloride, heated at 95°C for 5 min and then gradually cooled to RT to allow annealing.

### *Controls*

In vitro translated p53 protein was used as a positive control in the DNA binding experiments. Briefly, the procedure was the following: the expression vector pGEMhp53wtB was linearized by digestion with *Bam*HI. Capped transcripts were prepared using T7 RNA polymerase (Promega) and then translated in reticulocyte lysates as recommended by the supplier (Promega). Successful translation was verified by Western blotting using antibody DO1. The SP-1 oligonucleotide was used as a p53-unrelated oligonucleotide competitor.

### *DNA binding assay*

Three microlitres of nuclear extracts (10 μg) was preincubated for 10 min with 100 ng of purified monoclonal antibody PAb421 to activate specific DNA binding of p53 protein. It has been shown that p53 binds non-specifically to DNA through the carboxy-terminal domain, locking p53 in a conformation that cannot bind its specific element. By adding PAb421, non-specific binding is inhibited and p53 adopts the specific DNA conformation (Halazonetis et al, 1993; Bayle et al, 1995). Furthermore, nuclear extracts from p53 null cells (Saos2) were added to the reaction, in order to get in vitro translated p53 to bind the p53RE (Funk et al, 1992). Subsequently, 1 ng of labelled p53RE in the presence of 500 ng of poly(dI)-poly(dC) as non-specific DNA competitor was added. All incubations took place on ice with the following binding conditions: 50 mM potassium chloride, 25 mM Hepes, pH 7.6, 5 mM DTT, 10 μg of leupeptin per ml, 0.05% Triton X-100 and 20% glycerol. Competition experiments were performed by preincubating PAb421-activated extracts with 50-fold molar excess of unlabelled oligonucleotides (p53RE or SP-1) or 100 ng of anti-p53 antibody DO1. Protein–DNA binding reactions were analysed on a 4% polyacrylamide gel. Gels were dried and exposed on radiograph film (RX Fuji) at –70°C.

### Transfection assays

### *Cell line and plasmids*

H1299 cells (a human non-small-cell lung carcinoma cell line that contains a homogeneous deletion of the *p53* gene) were obtained from the American Type Culture Collection (Rockville, MD, USA) and maintained in RPMI-1640 medium supplemented with 10% fetal calf serum in a 37°C incubator containing 5% carbon dioxide. Reporter plasmid pGL2hmdm-HX-luc was constructed, as described previously (Zauberman et al, 1995*a*), by excising the *Hin*dIII–*Xho*I fragment from the amplified *hMDM2* DNA, and subcloning it into pGL2-Basic (Promega). The representative p53 mutants, V157F, R209T (group A), V173E (group B) and R273H (group C), were generated by site-directed mutagenesis of the parental wtp53 vector pCB6p53wt. The pCB6p53wt plasmid contains the published *p53* cDNA sequence (Matlashewski et al, 1984; Zakut-Houri et al, 1985).

### *Site-directed mutagenesis*

The site-directed mutagenesis procedure we followed was based on the method of Deng and Nickoloff (1992). This method works by simultaneously annealing two oligonucleotide primers to one strand of a denaturated double-strand plasmid. One primer introduces the desired mutation (mutagenic primer), whereas the other eliminates a unique restriction site of the plasmid for the purpose of selection (selection primer). After synthesis of a hybrid plasmid and a primary selection by digesting with the appropriate restriction enzyme, the

© Cancer Research Campaign 1998

**Figure 1** Analysis of the interaction between mutant p53 protein, R209T, produced in sample 5 and p53 responsive element (p53RE) of the *MDM2* gene. (**A**) Position of p53 mutation in case 5, obtained by direct sequencing of the nested-PCR product of exon 6 (arrow). The mutation changes codon 209 from TCT (R) to TGT (T) (antisense strand). The left-hand sequence represents wild-type DNA. N, normal; T, tumour. (**B**) Electrophoretic mobility shift assay showing sequence-specific binding of the mutant p53, R209T, to the p53RE of the *MDM2* gene. Lane 1, mutant p53 R209T binds with the specific DNA, in the presence of PAb421, and forms a retarded band (middle arrow). The specificity of the band was demonstrated with the following reactions. Lane 2, 50-fold molar excess of unlabelled *MDM2*-p53RE probe, in the presence of PAb421, markedly diminished the retard band. Lane 3 (control), in vitro translated wild-type p53 was incubated with labelled p53-RE, in the presence of PAb421, and produced a retard band at the same height as R209T.*: In the last reaction we added nuclear extracts from the p53 null cell line Saos2 to achieve p53 specific binding (Funk et al, 1992). Lane 4, the addition of anti-p53 antibody DO-1, in the presence of PAb421, super-shifted the retard band indicating the presence of p53 in the protein-p53 complex (Niewolik et al, 1995) (upper arrow). (**C**) Western blotting with antibody 1B10 showing the MDM2 protein isoform p76/74 expressed in sample 5 (lane 5). Lanes 1–4 represent MDM2 proteins produced from cases included in our previous work (Gorgoulis et al, 1996*b*)

hybrid plasmid is transformed into a *mut*S *E. coli* strain defective in a mismatch repair. This step is followed by an amplification procedure that results in a large pool of mutated and parental plasmids. The isolated DNA is then subjected to a series of selective restriction enzyme digestions that destroy the parental plasmid. A final transformation using the thoroughly digested DNA will result in the recovery of the desired mutant plasmid. In the present study, we used a selective primer which eliminates the unique *NdeI* restriction site and introduces an *NcoI* restriction site in the parental plasmid pCB6p53wt, and mutagenic primers:

for V157F: 5′-GGCACCCGC*TTC*CGCGCCATGGC-3′
for R209T: 5′-TTGGATGAC*ACA*AACACTTTTCGAC-3′
for V173E: 5′-GACGGAGGTT*GTG*AGGCGCTGCC-3′
and
for R273H: 5′-GCTTTGAGGTG*CAT*GTTTGTGCCTG-3′

 © Cancer Research Campaign 1998

**Table 2** Transcriptional transactivation by mutant p53 proteins with pGL2hmdm-HX-luc reporter

| Transfected DNA | Change | | Per cent of pGL2hmdm-HX-lu activity |
|---|---|---|---|
| | from: | to: | |
| pCB6 (control) | – | | 15 |
| pCB6p53wt (wtp53) | – | 100 | |
| pCB6p53mt V157F (mtp53-V157F) | GTC | TTC | 72 |
| pCB6p53mtR209T (mtp53-R209T) | AGA | ACA | 21 |
| pCB6p53mtV173E (mtp53-V173F) | GTG | GAG | 14 |
| pCB6p53mtR273H (mtp53-R273H) | CGT | CAT | 12 |
| wtp53/mtp53-R273H: 1/1 | – | | 40 |
| wtp53/mtp53-R273H: 1/4 | – | | 61 |

The *E. coli* bacterial strains we used for the first and final transformation were BMH71-18 *mut*S and XL1-Blue respectively. Successful mutagenic procedure was verified by sequencing.

### *Transient transfections and luciferase assay*

One microgram of the reporter plasmid pGL2hmdm-HX-luc was mixed with 1 μg of the expression vectors encoding wt p53, mt p53 V157F and R209T (group A), mt p53 V173L (group B), mt p53 R273H (group C) and control vector, pCB6, respectively, in RPMI-1640 medium. An aliquot (10 μg) of diluted in RPMI (1:100) Transfectam reagent (Promega) was immediately added to the plasmid mixture. The Transfectam reagent is a synthetic, cationic lipopolyamine with high affinity for DNA, coating it with a cationic lipid layer that facilitates binding to the cell membrane. The Transfectam/plasmid mixture was then added directly to 5 × $10^5$ exponentially growing H1299 cells in RPMI-1640 medium (without serum). The cells were incubated at 37°C with 5% carbon dioxide for 48 h. The transfected cells were collected, washed with phosphate-buffered saline, and lysed in the lysis buffer provided with the luciferase kit (Promega). Transcriptional activity was measured using a luminometer (Pharmacia LKB Nuclear, Gaithersburg, MD, USA). Successful transfection was verified by Western blotting using antibody DO1.

**Figure 2** Effects of the p53 mutants on the transcriptional activity of the luciferase reporter gene downstream of the *MDM2*-p53RE of the pGL2hmdm-HX-luc plasmid. The total amount of DNA transfected in each experiment was 2 μg (1 μg of wt and/or mt p53 and 1 μg of the reporter plasmid). Activity was expressed as a percentage of that induced by 1 μg of wt p53. Results shown are an average of three independent transfection experiments

### RESULTS

### Immunohistochemical and molecular profile of p53 and MDM2 proteins in the selected bronchogenic carcinomas (Table 1)

The data concerning the characteristics of p53 and MDM2 proteins in the selected cases of groups A (cases 1–5), B (cases 6–8) and C (cases 9–12) are presented in detail in Table 1. All the mutations were single-base substitutions and missense (six transversions and four transitions) (Figure 1A) with the exception of a frame-shift mutation detected in case 9. In cases 1–4, they were located in the β sandwich of the p53 protein (S4, S8, S4 and S9, respectively, Cho et al, 1994) that forms a scaffold to support the DNA-binding domain. This region of p53 is less conserved than the DNA-binding domain and possesses less than 1% of all p53 mutations. Mutations detected in this area are known as conformational. In specimen 5, the mutation was also conformational with the difference that it occurred in a β-turn connecting S6 and S7 (Cho et al, 1994). The mutations in group B and in specimens 10 and 11 of group C were located in the DNA-binding domain and are classified as contact mutants (Cho et al, 1994). This region accounts for the majority of all p53 mutations. Notably, in case 12, although IHC and immunoblotting showed p53 overexpression, direct sequencing did not indicate any alteration in the *p53* gene, therefore it expressed the wt p53 protein. Immunoblotting analysis, in the specimens that expressed MDM2, revealed various patterns of three MDM2 protein isoforms of approximately 90 kD (p90), 76/74 kD (p76/74), and 57 kD (p57) (Figure 1C). D-PCR analysis showed that overexpression of the MDM2 proteins was due to increased transcription and not to *MDM2* gene amplification.

### DNA binding properties of mutant p53 proteins (Table 1)

#### *Group A*

DNA-binding experiments revealed in cases 1, 2 and 5 a common retard band (Figure 1B, lane 1). This band was supershifted when anti-p53 antibody DO1 was included in the reaction mixture (Figure 1B, lane 4) and abolished in the presence of excess unlabelled p53RE (Figure 1B, lane 2). Competition with molar excess of SP-1 did not abolish the band. These findings indicated the presence of p53 in the protein–DNA complex (Niewolik et al,

© Cancer Research Campaign 1998

**Figure 3** Schematic representation of the mechanisms we suggest that deregulate the p53/MDM2 autoregulatory feedback loop in a subset of bronchogenic carcinomas. Comparison with the p53/MDM2 autoregulatory feedback loop that exists in normal cells. (**A**) In a normal cell not under stress wild-type p53 (wt p53) protein is expressed at very low levels and plays little role in the normal cell cycle (Vogelstein et al, 1992). The *MDM2* gene is very weakly stimulated by wt p53 and the low levels of the MDM2 protein p90 are produced mainly due to the basal activity of the P1 promoter (Zauberman et al, 1995*a*). (**B**) In a normal cell under stress (DNA damage, hypoxia, metabolic changes and immunological response), the *MDM2* gene is expressed only when wt p53 has activated the targets that mediate its 'protective' action (e.g. *gadd45, waf-1/cip-1*). When the cell contains relatively large amounts of wt p53, it binds transiently the p53 RE of *MDM2*, triggering the production of MDM2 protein p90 that eventually terminates p53 cycle arrest mediated signal (reviewed by Gottlieb and Oren, 1996; Ko and Prives, 1996; Oren and Prives, 1996; Levine, 1997). Furthermore, p90 promotes the cell cycle by inactivating the oncosuppressor protein Rb (Xiao et al, 1995) and enhancing the activity of the S-phase inducing transcription factor E2F1 (Martin et al, 1995). (**C**) In the environment of a lung carcinoma cell the following oncogenic mechanisms may possibly develop: 1. Constantly elevated levels of mutant p53 proteins permanently stimulate the MDM2 gene (directly or indirectly, see Discussion) producing high levels of MDM2 mRNA. Excess MDM2 mRNA is probably responsible, via a complex dose-dependent mechanism (Zauberman et al, 1995a; Barak et al, 1994), for the production of various MDM2 proteins, several of which do not form an autoregulatory loop with p53 (Gorgoulis et al, 1996b). The overexpressed p90 isoform positively augments cell proliferation by interacting with E2F1. The other MDM2 proteins, probably, add novel oncogenic properties to the cell (Shigalas et al, 1996). 2. Inactive mt p53 proteins exert a dominant positive effect on the transcription activity on the wt p53 protein. 3. Alterations at the P1 promoter may activate a p53-independent mechanism of MDM2 overexpression. 4. Mutant p53 has the ability to complex with the MDM2 p90 isoform enhancing probably the tumorigenic potential (Momand et al, 1992)

1995) and revealed that *p53* mutants V157F, Y234C and R209T (Fig. 1A) maintained the wild-type p53 property to bind the *MDM2*-p53RE. Interestingly, the substituted amino acids in the first two specimens possessed similar residual (-R) groups. In case 1, the hydrophobic non-polar *Val* was replaced by *Phe* and in case 2 the hydrophilic uncharged *Tyr* was replaced by *Cys*. In contrast, in case 5, at position 209, the hydrophilic uncharged amino acid *Thr* took the place of the positive charged *Arg*. Finally, mutants R158G (case 3) and E258G (case 4) showed no DNA binding activity. The substituted amino acids in these specimens also belonged to different classes. More specifically, the positively charged *Arg* and the negatively charged *Glu* were replaced by the uncharged hydrophilic *Gly*.

### *Group B*

The p53 mutants of this group, A138D, A138S and V173E, did not interact with the *MDM2*-p53RE. It is notable that the first two cases possessed a point mutation at the same codon that changed the hydrophobic amino acid *Ala* to the negatively charged *Asp* and hydrophilic uncharged *Ser* respectively. The third member (case 8) had a similar substitution as in case 6; the hydrophobic amino acid *Val* was replaced by the negatively charged *Glu*.

### *Group C*

As we expected, mutant p53 in case 9, which had a frameshift mutation at codon 195, did not bind the p53RE, whereas sample 12, which produced the wt p53, interacted strongly with the

*© Cancer Research Campaign 1998*

element. Nuclear extracts from the remaining specimens 10 and 11, which contained mutants R273H and C275Y, showed strong binding with the p53RE. Both mutations produced amino acid substitutions with similar (-R) residual groups. However, it should be mentioned that the last two samples maintained the wt p53 allele and therefore we cannot exclude the possibility, as we will discuss later, of DNA binding being achieved by p53 oligomers containing wt and mt p53 proteins (Stenger et al, 1992).

### Relationship between DNA binding abilities of p53 mutants and *MDM2* mRNA and protein levels (Table 1)

All the mutants that did not alter the net charge of the p53 molecule (V157F, Y234C, R273H and C275Y) showed DNA-binding activity, increased levels of *MDM2* mRNA and MDM2 protein overexpression. Only mutant R209T, which did not follow the above rule, demonstrated similar findings. In contrast, three of the four mutants (A138D, A138S and V173E), that altered the net charge of the molecule, did not interact with the p53RE and were not accompanied by *MDM2* mRNA and MDM2 protein expression. In the remaining specimens, 3 and 4, *MDM2* mRNA and MDM2 proteins were overexpressed in spite of the fact that the mutants R158G and E258G showed no binding activity.

### Transient transfection assays (Table 2, Figure 2)

To determine whether specific binding activity was correlated with transactivation of the *MDM2*-p53RE, we constructed four representative p53 mutants: two from group A, V157F and R209T, and one each from group B, V173E, and C, R273H, and co-transfected them with the luciferase reporter plasmid pGL2hmdm-HX-luc, which contains the *MDM2*-p53RE, into the p53-null human non-small-cell lung carcinoma cell line H1299. Successful transfection was verified with Western blot analysis of the mt p53 proteins. In addition, in each experiment along with the p53 mutant plasmid the wt p53 vector pCB6p53wt and the pCB6 vector, as positive and negative control, respectively, were co-transfected with the reporter plasmid. The activity of the luciferase gene with each mutant was tested with the wt p53 vector set at 100%. The experiments were carried out independently three times with the same results:

#### *Group A*

Mutant p53 V157F enhanced the luciferase activity of pGL2hmdm-HX-luc at about 72% of the wt p53 vector, whereas mutant R209T, which was detected in a small-cell carcinoma and showed a strong interaction with the responsive element, failed to stimulate it in the H1299 cell culture (Table 2, Fig. 2). These data and those of others (see Discussion) suggest that the biological and biochemical functions of p53 mutants are dependent on the specific missense mutations acquired in the p53 gene and probably the cellular environment in which they act.

#### *Group B*

As we expected mt p53 V173E, which failed to bind the p53RE in the bandshift assay, was also unable to transactivate it.

#### *Group C*

As we mentioned, p53 mutant R273H, in case 10, was accompanied by the wt p53 allele. Thus, we could not exclude the possibility that DNA binding was achieved by heterotetramers containing the normal and mt p53 proteins (Stenger at al, 1992). Therefore, to clarify the picture we performed two kinds of experiments: first, we co-transfected the mt p53 plasmid alone with the reporter plasmid and then the mt with the wt p53 at ratios 1:1 and 4:1. The total amount of transfected mutant and wt p53 plasmid DNA was always 1 μg. As shown in Table 2 and Figure 2 mutant R273H was unable alone to activate the *MDM2*-p53RE. However, when mt and wt p53 plasmids were mixed at ratios 1:1 and 4:1 and co-transfected with the reporter plasmid the luciferase activity was increased from basal levels to 40% and 61% respectively. This result, as we will discuss later, suggests a dominant positive effect of inactive p53 mutant R273H over wt p53.

### DISCUSSION

In the present work, we examined the ability of mutant p53 proteins derived from selected lung carcinomas to bind the *MDM2*-p53RE. The findings were correlated with *MDM2* mRNA and protein levels. Furthermore, we constructed four of these p53 mutants and studied their transactivation abilities by co-transfecting them with a reporter plasmid carrying the *MDM2*-p53RE into the *p53* null non-small-cell lung carcinoma cell line (NSCLC) H1299.

Selection was based on certain criteria that allowed us the study of a homogeneous cancerous population as far as p53 and MDM2 expression is concerned. The cases were placed in three groups (see Materials and methods).

DNA-binding experiments revealed that three out of the five p53 mutants of group A (cases 1, 2 and 5) retained their DNA-binding properties. Their mutations were detected at codons 157 (V→F), 234 (Y→C) and 209 (R →T). As all these cases showed overexpression of various *MDM2* gene products due to increased transcription and not to *MDM2* gene amplification, we suggested that certain *p53* missense mutations, apart from stabilizing p53 (Zambetti and Levine, 1993; Gottlieb and Oren, 1996), generate proteins that may maintain several of the wt p53 properties and possibly acquire novel ones. The molecular basis of the effect that missense mutations have on the behaviour of p53 is not clear. One possible explanation relies on the charge difference they introduce into the p53 amino acid sequence. Interestingly, the substitutions in p53 mutants V157F and Y234C were conservative and thus did not alter the net charge of molecule. Similar were the findings of Zhang et al. (1993a), Park et al. (1994) and Niewolik et al. (1995). In their studies 'hotspot' p53 mutants, derived from different cell lines, were tested for their ability to bind the original consensus p53 DNA binding site (p53 CON) (Funk et al, 1992). The mutants with DNA-binding activity had substitutions that retained the net charge of p53 and, furthermore, they maintained the ability to transactivate the p53 CON. In contrast, although the amino acid change in p53 mutant R209T was non conservative, it interacted with the *MDM2*-p53RE strongly. A probable interpretation could reside on the secondary structure that this substituation participates and the context in which this structure folds. Thus, position 209 lies in a β-turn of the β-sandwich region of p53 and the relative frequency of occurrence for *Thr* in β-turn is higher than Arg (Creighton, 1984). It is therefor possible that this particular substitution does not disrupt the secondary structure of the β-turn and the conformation of p53 protein generally. Crook et al. (1994) studying the growth and transformation suppression functions of a large series of mutants observed that two tumour-derived point mutants, p53 R175P and R181L, activated the *MDM2*-p53 RE as

© Cancer Research Campaign 1998

wt p53 did in primary rat embryo fibroblasts and Saos-2 cells. They detected, by Western blotting, elevated levels of several MDM2 protein isoforms. Both mutations were non-conservative and concerned the highly sensitive in alterations loop L2 and α-helix H1 of the DNA binding domain of p53 (Cho et al, 1994). Interestingly, both substitutions have a higher relative frequency of occurrence in the aforementioned secondary structures than *Arg* (Creighton, 1984). Three additional reports by Chen et al (1993), Ory et al (1994) and Kawamura et al (1996) provide evidence that could support both sides. These groups studied the biological properties of several p53 mutants and observed a diversity in their functions making the picture of mutants p53 behaviour much more complex. Our opinion is that net charge difference is an important factor in predicting the binding behaviour of mt p53 as all the mutants in the present work that did not associate with the responsive element (mutants R158G and E258G from group A and all the mutants of group B) had substitutions that altered its net charge. However, steric effects, hydrophobicity and interaction with other proteins may also play a role.

The binding affinity of the mutants we examined was similar to that of the in vitro translated wt p53 used as control (Figure 1B, lane 3). Niewolik et al. (1995) examining the affinity of p53 mutant proteins for the *MDM2*-p53RE, noticed a more than ten-fold reduction in their binding activity. These differences may reflect either a conformational and/or cell type specific effect. Several investigators have shown that wt and mt p53 may regulate certain promoters in a specific cell type manner (Grinsberg et al, 1991; Santhanam et al, 1991; Chin et al, 1992; Subler et al, 1992; Mack et al, 1993; Haupt et al, 1996; Rowan et al, 1996); for example the PCNA promoter was not repressed by wt p53 in the study of Mack et al (1993) using cervical epithelium cells but the same promoter was repressed by wt p53 in the study of Subler et al (1992) using Hela or Vera cells. Rowan et al. (1996) demonstrated that mutant p53 R175P could induce growth arrest but not apoptosis in Saos-2 and H1299 cells, whereas HeLa and H358 cells retained some apoptotic function after R175P expression. This cell-type effect may be due to specific cellular co-factors that couple with the activated form of p53, thus modulating its function.

The p53 mutant proteins in specimens 3 and 4, which also showed MDM2 overexpression, did not bind the *MDM2*-p53RE. It seems that in these cases MDM2 overexpression is due either to a p53-independent mechanism or to an indirect mode of action of mt p53. The basis for both mechanisms possibly relies on the way *MDM2* gene is organized. The *MDM2* gene possesses two promoters P1 and P2 (Figure 3). P1 is a p53-independent promoter located upstream from exon 1 and is active at basal constitutive levels and P2 is the p53-dependent promoter located in intron 1 and contains the p53 RE accompanied by the general transcription element TATA box (Zauberman et al, 1995*a*). In the first case, the p53-independent mechanism of *MDM2* overexpression is possibly activated by alterations in the regulatory elements of P1. Although our experiments did not reveal *MDM2* gene amplification, we cannot exclude the possibility of regional gene alterations (e.g. deletion of silencer elements), which could participate in this putative mechanism. In the second, mutant p53 may favour transcription in a general way. This assumption is based on several observations. It has already been shown that the N-terminus of wt p53 directly interacts with TBP (TATA-binding protein) and other general transcription factors (Martin et al, 1993; reviewed by: Zambetti and Levine, 1993; Gottlieb and Oren, 1996; Ko and Prives, 1996; Oren and Prives, 1996; Levine, 1997). The presence of p53 binding sites near the TATA element possibly allows the nucleation on these promoters, thus enhancing transcriptional activity. In the case of mt p53 defective in DNA binding, the interaction with these transcription factors may be preserved and a general activation of P2 may be achieved. In accordance with this speculation is the finding of Martin et al (1993), who observed that mt p53 V218G, which was bound with TBP, did activate a promoter with p53 binding sites in vivo, although the DNA binding activity of the mutant was abolished.

The findings in group A were further enforced by the observations in group B. The mutants in this group (A138D, A138S and V173E) did not bind the *MDM2*-p53RE and were not accompanied by MDM2 expression. Codon 138 is localized near the transcriptional activation domain of p53. The integrity of the transactivation domain is shown to be critical for 'gain of function' p53 mutants (Lin et al, 1995). Possibly these mutants, apart from losing their DNA binding properties, suffer a conformational change in the N-terminus domain that interferes with their ability to interact with factors of the transcription machinery. Codon 173 is involved in stabilizing interactions between loops L2 and L3 of p53 protein. Mutations at this region are considered among the most disruptive for p53 functions (Prives, 1994).

The four samples included in the heterogeneous group C provided additional evidence concerning the functions of mt and wt p53. In sample 9, p53 did not show binding activity because the frameshift mutation at codon 195 altered its reading frame producing either a non-functional mRNA or an altered protein with unknown functions. MDM2 overexpression in sample 12 is possibly caused by constant stimulation of the *MDM2* gene by the accumulated wt p53. Normally wt p53 has a short half-life (Zambetti and Levine, 1993; Gottlieb and Oren, 1996), but cellular stress (Hall et al, 1996) and certain proteins, including MDM2, can induce stabilization of the protein (Momand et al, 1992). Recently, Haupt et al (1996) demonstrated that MDM2 overexpression in H1299 cells resulted in effective protection from apoptosis. Loss of p53 induced apoptosis even without a concomitant loss of $G_1$ arrest could contribute to cancer progression. This mechanism of action could exist in our case as well. Finally, p53 mutants R273H and C275Y behaved in a similar manner as mutants in samples 1, 2 and 5. As these cases retained the wt p53 allele we hypothesized that MDM2 protein isoform overexpression was achieved either by the mutant in a wt p53-independent manner (wt p53 allele non-functional) or by a dominant positive effect of the mt p53 on the functional wt p53.

To examine the credibility of the hypotheses we have suggested above, we constructed four of these p53 mutants, two from group A, V157F and R209T, and one from group B, V173E, and C, R273H, co-transfected them with a luciferase reporter plasmid carrying the *MDM2*-p53RE, in the *p53* null H1299 cell line and studied their transactivation abilities. Mutant V157F enhanced luciferase expression at about 72% (Figure 2, Table 2) of the wt p53 vector conforming our initial speculation that, MDM2 overexpression in certain lung carcinomas may be due to a 'gain of function' mutant p53 phenotype (Gorgoulis et al, 1996*a*,*b*). In contrast, mutant R209T, which showed a strong interaction with the responsive element, failed to stimulate it. This finding may be due to determinants of the H1299 cellular context that altered the function of the protein. The effect of the cellular environment on wt and mt p53 activities, as we mentioned, is gradually becoming clearer (Oren and Prives, 1996). In the present case, R209T was detected in a small-cell lung carcinoma (SCLC), whereas H1299 is

 © Cancer Research Campaign 1998

a non-small-cell lung carcinoma (NSCLC)-derived cell line. Although from a histogenetic point of view all histological variants of bronchogenic carcinoma have a common origin, SCLCs are much more aggressive and are treated differently than NSCLCs (Mackay et al, 1991). Whereas transfection with V173E, as we expected, showed no surprises (Figure 2, Table 2) transfection with R273H showed some interesting results. More specifically, when R273H was introduced in the cells alone with the reporter plasmid no luciferase activity was observed but when R273H and wt p53 expression vectors were mixed in a 1:1 and 4:1 ratio, luciferase activity was increased gradually from basal levels to 40% and 61% respectively (Figure 2, Table 2). This finding conformed the hypothesis we stated and suggested that dominant negative inhibition by mt p53 is not the rule and that certain p53 mutants, depending on the binding element and possibly the appropriate environment, have a 'positive effect' on wt p53 function. Similar were the results of Zhang et al (1993*b*) using the p53CON element. In their study, they proposed that the relative ratio of mt to wt p53 in the heterotetramers they form is important for transactivation.

Taking together the above data, we propose the following hypothesis (Figure 3): in a normal cell under stress, *MDM2* can be transactivated only when wt p53 has activated the targets that mediate its action (e.g. *gadd45*, *waf-1/cip-1*). When the cell contains relatively large amounts of wt p53, it binds transiently the p53RE of *MDM2*, triggering *MDM2* expression that eventually terminates p53 cycle arrest mediated signal. In contrast, constantly elevated levels of mt or wt p53 in lung carcinoma cells permanently stimulate the *MDM2* gene (directly or indirectly), producing high levels of *MDM2* mRNA. Excess *MDM2* mRNA is probably responsible, via complex mechanisms (Barak et al, 1994; Zauberman et al, 1995*a*), for the production of various MDM2 proteins, several of which do not participate in the autoregulatory loop with p53 (Gorgoulis et al, 1996*b*). These proteins possibly add novel oncogenic properties to the cell (Sigalas et al, 1996).

In conclusion, the present study suggests that deregulation of the p53/MDM2 autoregulatory feedback loop, in a subset of lung carcinomas, may be due to either a 'gain of function' mt p53 phenotype or to a dominant positive effect of certain p53 mutants on the wt p53 protein.

### ACKNOWLEDGEMENTS

The authors wish to thank Professor M Oren for his kind suggestions in the course of the study and for providing the expression vector pCB6p53wt, the reporter plasmid pGL2hmdm2-HX-luc and the H1299 cell line. We also wish to thank Dr PP Sfikakis and Dr P Panagiotidis for technical support, Dr F Mavrommatis and N Rezaie for their helpful critisism in the manuscript and, also Dr DP Lane for providing antibody DO1.

### REFERENCES

Barak Y, Gottlieb E, Juven-Gershon T and Oren M (1994) Regulation of mdm2 expression by p53: alternative promoters produce transcripts with non identical translation potential. *Genes Dev* **8**: 1739–1749

Bayle JH, Elenbaas B and Levine AJ (1995) The carboxyl-terminal domain of p53 protein regulates sequence-specific DNA binding through its non-specific nucleic acid-binding activity. *Proc Natl Acad Sci USA* **92**: 5729–5733

Buckbinder L, Talbott R, Valesco-Miguel S, Takenaka I, Faha B, Seizinger BR and Kley N (1995) Induction of the growth inhibitor IGF-binding protein 3 by p53. *Nature* **377**: 646–649

Bueso-Ramos CE, Yang Y, deLeon E, McCown P, Stass SA and Albitar M (1993) The human MDM2 oncogene is overexpressed in leukemias. *Blood* **82**: 2617–2623

Bueso-Ramos CE, Manshouri T, Haidar M, Huh YO, Keating MJ and Albitar M (1995*a*) Multiple patterns of MDM2 deregulation in human leukemias: Implications in leukemogenesis and prognosis. *Leuk Lymph* **17**: 13–18

Bueso-Ramos CE, Yang Y, Manshouri T, Feltz L, Ayala A, Glassman AB and Albitar M (1995*b*) Molecular abnormalities of MDM-2 in human sarcomas. *Int J Oncol* **7**: 1043–1048

Bueso-Ramos CE, Manshouri T, Yang Y, McCown P, Ordonez NG, Sneige N and Albitar M (1996) Abnormal expression of multiple MDM-2 proteins in breast carcinomas. *Breast Cancer Res Treat* **85**: 29–40

Chen JY, Funk DK, Wright WE, Shay JW and Minna JD (1993) Heterogeneity of transcriptional activity of mutant p53 proteins and p53 DNA target sequences. *Oncogene* **8**: 2159–2166

Chen X, Bargonetti J and Prives C (1995) p53 through p21 (WAF1/CIP1), induces cyclin D1 synthesis. *Cancer Res* **55**: 4257–4263

Chin KV, Ueda K, Pastan I and Gottesman MM (1992) Modulation of activity of the promoter of the human MDR1 gene by Ras and p53. *Science* **255**: 459–462

Cho Y, Gorina S, Jeffrey PD and Pavletich NP (1994) Crystal Structure of a p53 Tumour Suppressor-DNA Complex: Understanding Tumorigenic Mutations. *Science* **265**: 346

Creighton TE (1984) *Proteins Structures and Molecular Properties* p. 235 WH Freeman:

Crook T, Marston NJ, Sara EA and Vousden KH (1994) Transcriptional activation by p53 correlates with suppression of growth but not transformation. *Cell* **79**: 817–827

Dameron KM, Volpert OV, Tainsky MA and Bouck N (1994) Control of angiogenesis in fibroblast by p53 regulation of thrombospontin-1. *Science* **265**: 1582–1584

Deb SP, Munoz RM, Brown DR, Subler MA and Deb S (1994) Wild-type human p53 activates the human epidermal growth factor receptor promoter. *Oncogene* **9**: 1341–1349

Deng EC and Nickoloff JA (1992) Site-directed mutagenesis of virtually any plasmid by eliminating a unique restriction site. *Anal Biochem* **20**: 81

Deffie A, Wu H, Reinke V and Lozano G (1993) The tumor suppressor p53 regulates its own transcription. *Mol Cell Biol* **13**: 3415–3423

Dittmer D, Parti S, Zambetti G, Chu S, Teresky AK, Moore M, Finlay C and Levine AJ (1993) Gain of function mutations in p53. *Nature Genet* **4**(1): 42–46

El-Diery WS, Tokino T, Velculescu VE, Levy DB, Parson R, Trent GM, Lin D, Merker WE, Kinz KW and Volgenstein B (1993) WAF1, a potential mediator of p53 tumor suppression. *Cell* **75**: 817–825

Fontana X, Ferrari Abbes M, Monticelli Namer M and Bussiere F (1994) Etude de l'amplification du gene *mdm-2* dans les tumeurs primitives du cancer du sein. *Bull Cancer* **81**: 587

Foulkes WD, Stamp GWH, Afzal S, Lalani N, McFarlane CP, Trowsdale J and Campbell IG (1995) MDM2 overexpression is rare in ovarian carcinoma irrespective of TP53 mutation status. *Br J Cancer* **67**: 551–559

Funk WD, Pak DT, Karas RH, Wright WE and Shay JW (1992) A transcriptonally active DNA-binding site for human p53 protein complexes. *Mol Cell Biol* **12**: 2866–2871

Gorgoulis V, Zoumpourlis V, Rassidakis G, Karameris A, Barbatis C, Spandidos DA and Kittas C (1995*a*) Molecular analysis of p53 gene in laryngeal premalignant and malignant lesions: p53 protein immunohistochemical expression is positively related to proliferating cell nuclear antigen labelling index. *Virchows Archiv* **426**: 339–344

Gorgoulis V, Sfikakis PP, Karameris A, Papastamatiou H, Trigidou R, Veslemes M, Spandidos DA, Sfikakis P and Jordanoglou J (1995*b*) Molecular and immunohistochemical study of class I growth factor receptors in squamous cell lung carcinomas. *Path Res Pract* **191**: 973–981

Gorgoulis V, Rassidakis G, Karameris A, Papastamatiou H, Trigidou R, Veslemes M, Rassidakis A and Kittas C (1996*a*) Immunohistochemical and molecular evaluation of the *MDM2* gene product in bronchogenic carcinoma. *Mod Path* **9**: 544–554

Gorgoulis V, Zoumpourlis V, Rassidakis G, Karameris A, Rassidakis A, Spandidos DA and Kittas C (1996*b*) A molecular and immunohistochemical study of the MDM2 protein isoforms and *p53* gene product in bronchogenic carcinoma. *J Path* **180**: 129–137

Gottlieb TM and Oren M (1996) p53 in growth control and neoplasia. *Biochim Biophys Acta* **1287**: 77–102

Greenblatt MS, Bennett WP, Hollstein M and Harris CC (1994) Mutations in the *p53* tumour suppressor gene: Clues to cancer etiology and molecular pathogenesis. *Cancer Res* **54**: 4855–4878

© Cancer Research Campaign 1998

Grinsberg D, Mechta F, Yaniv M and Oren M (1991) Wild-type p53 can down-modulate the activity of various promoters. *Proc Natl Acad Sci USA* **88**: 9979–9983

Halazonetis TD and Kandil AN (1993) Conformational shifts propagate from the oligomerization domain of p53 to its tetrameric DNA binding domain and restore DNA binding to select p53 mutants. *EMBO J* **12**: 5057–5064

Hall P, Meek D and Lane DP (1996) p53-integrating the complexity. *J Path* **180**: 1–5

Haupt T, Barak Y and Oren M (1996) Cell type specific inhibition of p53-mediated apoptosis by mdm2. *EMBO J* **15**: 1596–1606

Hinds P, Finlay C and Levine AJ (1989) Mutation is required to activate the p53 gene for cooperation with the ras oncogene and transformation. *J Virol* **63**: 739–746

Hsiao M, Low J, Dorn E, Ku D, Pattengale P, Yeargin J and Haas M (1994) Gain of function mutations of the p53 gene induce lympohematopoietic metastasic potential and tissue invasiveness. *Am J Pathol* **145**: 702–714

Kastan MB, Zhan Q, El-Diery WS, Carrier F, Jacks T, Walsh WV, Plunkett BS, Volgenstein B and Fornance AJ Jr (1992) A mammalian cell cycle checkpoint pathway utilizing p53 and GADD45 is defective in ataxia-telegiectasia. *Cell* **71**: 587–597

Kawamura M, Yamashita T, Segawa K, Kaneuchi M, Shindoh M and Fujinaga K (1996) The 273rd codon mutants of p53 show growth modulation activities not correlated with p53-specific transactivation activity. *Oncogene* **12**: 2361–2367

Ko LJ and Prives C (1996) p53: puzzle and paradigm. *Genes Dev* **10**: 1054–1072

Levine AJ (1997) p53, the cellular gatekeeper for growth and devision. *Cell* **88**: 323–331

Lin J, Teresky AK and Levine AJ (1995) Two critical hydrophobic amino acids in the N-terminal domain of the p53 protein are required for the gain of function phenotypes of human p53 mutants. *Oncogene* **10**: 2387–2390

Mack DH, Vartikar J, Pipas JM and Laimins LA (1993) Specific repression of TATA-mediated but not initiator-mediated transcription by wild-type p53. *Nature* **363**: 281–283

Mackay B, Lukeman JM and Ordoñez N (1991) *Tumors of the Lung* WB Saunders: Philadelphia, USA

Martin DW, Munoz RM, Subler MA and Deb S (1993) p53 binds to the TATA-binding protein-TATA complex. *J Biol Chem* **268**: 13062–13067

Martin K, Trouche D, Hagemeier C, Sorensen TS, La Thangue NB and Kouzarides T (1995) Stimulation of E2F1/DP1 transcriptional activity by MDM2 oncoprotein. *Nature* **375**: 691–694

Matlashewski G, Lamb P, Pim D, Peacock J, Crawford L and Benchimol S (1984) Isolation and characterization of a human p53 cDNA clone: expression of the human p53 gene. *EMBO J* **3**: 3257–3262

Michalovitz D, Halevy O and Oren M (1991) p53 mutations: gain or losses? *J Cell Biochem* **45**: 22–29

Milner J, Medcalf EA and Cook AC (1991). Tumor suppressor p53: analysis of wild type and mutant p53 complexes. *Mol Cell Biol* **11**: 12–19

Miyashita T and Reed JC (1995) Tumor suppressor p53 is a direct transcriptional activator of the human bax gene. *Cell* **80**: 293–299

Momand J, Zambetti GP, Olson DC, George D and Levine AJ (1992) The MDM2 oncogene product forms a complex with the p53 protein and inhibits p53-mediated transactivation. *Cell* **69**: 1237–1245

Morris GF, Bischoff JR and Mathews MB (1996) Transcriptional activation of the human proliferating-cell nuclear antigen promoter by p53. *Proc Natl Acad Sci* **93**: 895–899

Muller BF, Paulsen D and Deppert W (1996) Specific binding of MAR/SAR DNA-elements by mutant p53. *Oncogene* **12**: 1941–1952

Niewolik D, Vojtesek B and Kovarik J (1995) p53 derived from human tumour cell lines and containing distinct point mutations can be activated to bind its consensus target sequences. *Oncogene* **10**: 881–890

Oca Luna RM, Wagner DS and Lozano G (1995) Rescue of early embryonic lethality in mdm2-deficient mice by deletion of p53. *Nature* **378**: 203–205

Olson D, Marechal V, Momand J, Chen J, Romochi C and Levine AJ (1993) Identification and characterization of multiple mdm2 proteins and mdm2 p53 protein complexes. *Oncogene* **8**: 2353–2360

Oren M and Prives C (1996) p53: upstream, downstream and offstream. Review of the 8th p53 workshop (Dundee, 5–9 July 1996). *Biochim Biophys Acta* 1288: R13–R19

Ory K, Legros Y, Auguin C and Soussi T (1994) Analysis of the most representative tumour-derived p53 mutants reveals that changes in protein conformation are not correlated with loss of transactivation or inhibition of cell proliferation. *EMBO J* **13**: 3496–3504

Osifchin NE, Jiang D, Ohtani-Fujita N, Fujita T, Carroza M, Kim S-J, Sakai T and Robbins PD (1994) Identification of a p53 binding site in the human retinoblastoma susceptibility gene promoter. *J Biol Chem* **269**: 6383–6389

Owen-Schaub LB, Zhang W, Cusack JC, Angelo LS, Santee SM, Fujiwara T, Roth JA, Deisseroth AB, Zhang W-W, Kruzel E and Radinsky R (1995) Wild type human p53 and a temperature sensitive mutant induce fas/APO1 expression. *Mol Cell Biol* **15**: 3032–3040

Park DJ, Nakamura H, Chumakov AM, Said JW, Miller CW, Chen DL and Koeffler HP (1994) Transactivational and DNA binding abilities of endogenous p53 in p53 mutant cell lines. *Oncogene* **9**: 1899–1906

Price BD and Park SJ (1994) DNA Damage Increases the Levels of MDM2 Messenger RNA in wtp53 Human Cells. *Cancer Res* **54**: 896–899

Prices C (1994) How loops, β sheets, and α helices help us to understand p53. *Cell* **78**: 543–546

Rowan S, Ludwig RL, Haupt Y, Bates S, Lu X, Oren M and Vousden H (1996) Specific loss of apoptotic but not cell cycle arrest function in a human tumor derived p53 mutant. *EMBO J* **15**: 827–838

Sambrook J, Fritsh EF and Maniatis T (1989) *Molecular Cloning. A Laboratory Manual*. Cold Spring Harbor, NY, Cold Spring Harbor Laboratory Press

Santhanam U, Ray A and Sehgal PB (1991) Repression of the interleukin 6 gene promoter by p53 and the retinoblastoma susceptibility gene product. *Proc Natl Acad Sci USA* **88**: 7605–7609

Shin TH, Paterson AJ and Kudlow JE (1995) p53 stimulates transcription from the human transforming growth factor-a promoter: a potential growth-stimulatory role for p53. *Mol Cell Biol* **15**: 4694–4701

Sigalas I, Calvert AH, Anderson JJ, Neal DE and Lunec J (1996) Alternatively spliced mdm2 transcripts with loss of p53 binding domain sequences: Transforming ability and frequent detection in human cancer. *Nature Med* **2**: 912–917

Spandidos DA, Zoumpourlis V, Zachos G, Toas SH and Halazonetis TD (1995) Specific recognition of a transcriptional element within the human H-ras proto-oncogene by the p53 tumour suppressor. *Int J Oncol* **7**: 1029–1034

Stenger JE, Mayr JA, Mann K and Tegtmeyer P (1992) Formation of stable homotetramers and multiples of tetramers. *Mol Carcinogen* **5**: 102–106

Subler MA, Martin DW and Deb S (1992) Inhibition of viral and cellular promoters by human wild-type p53. *J Virol* **66**: 6164–6170

Vogelstein B and Kinzler KW (1992) p53 Function and Dysfunction. *Cell* **70**: 523–526

World Health Organization (1982) The World Health Organization histologic typing of lung tumours. *Am J Clin Path* **77**: 123–136

Wu X, Bayle JH, Olson D and Levine AJ (1993) The p53-mdm2 autoregulatory feedback loop. *Genes Dev* **7**: 1126–1132

Xiao ZX, Chen J, Levine AJ, Modjtahedi N, Xing J, Sellers WR and Livingston DM (1995) Interaction between the retinoblastoma protein and the oncoprotein MDM2. *Nature* **375**: 694–698

Zakutt-Houri R, Bienz-Tadmor B, Givol D and Oren M (1985) Human p53 cellular tumour antigen: cDNA sequence and expression in COS cells. *EMBO J* **4**: 1251–1255

Zambetti GP and Levine AJ (1993) A comparison of the biological activities of wild-type and mutant p53. *FASEB J* **7**: 855–865

Zauberman A, Flusberg D, Haupt Y, Barak Y and Oren M (1995*a*) A functional p53-responsive intronic promoter is contained within the human mdm2 gene. *Nucleic Acids Res* **23**: 2584–2592

Zauberman A, Lubp A and Oren M (1995*b*) Identification of p53 target genes through immune selection of genomic DNA: The cyclin G gene contains two distinct p53 binding sites. *Oncogene* **10**: 2361–2366

Zhang W, Funk WD, Wright WE, Shay JW and Deisseroth AB (1993*a*) Novel DNA binding of p53 mutants and their role in transcriptional activation. *Oncogene* **8**: 2555–2559

Zhang W, Shay JW and Deisseroth A (1993*b*) Inactive p53 mutants may enhance the transcriptional activity of wt p53. *Cancer Res* **53**: 4772–4775

Zhang W, Randhawa GS, Gau J-P, Shay JW and Deisseroth AB (1995) The first intron of human H-ras is regulated by p53: Mediation of specific activation by a p53-binding element. *Int J Oncol* **7**: 1021–1028

 © Cancer Research Campaign 1998